# 1-Allyl-3,3-diphenyl­indolin-2-one

**DOI:** 10.1107/S1600536808009446

**Published:** 2008-04-16

**Authors:** S. Nirmala, E. Theboral Sugi Kamala, L. Sudha, A. R. Naresh Raj, C. A. M. A. Huq

**Affiliations:** a Department of Physics, Easwari Engineering College, Ramapuram, Chennai 600 089, India; b Department of Physics, SRM University, Ramapuram Campus, Chennai 600 089, India; c Postgraduate Research, Department of Chemistry, New College, Chennai 600014, India

## Abstract

In the title compound, C_23_H_19_NO, the oxindole residue is essentially planar and is almost perpendicular to the phenyl rings [dihedral angles = 72.1 (6)  and 77.6 (6)°]. The mol­ecular packing is stabilized by C—H⋯O hydrogen bonds and C—H⋯N inter­actions.

## Related literature

For related literature, see: Bandini *et al.* (2005 ▶); Florin *et al.* (1980 ▶); Govind *et al.* (2003 ▶); Rajeswaran *et al.* (1999 ▶); Ramirez & Garcia-Rubio (2003 ▶).
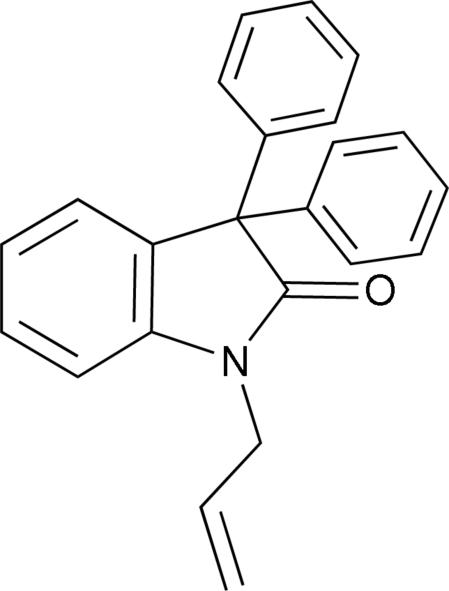


## Experimental

### 

#### Crystal data

C_23_H_19_NO
*M* *_r_* = 325.39
Orthorhombic, 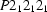

*a* = 8.8449 (3) Å
*b* = 12.3879 (4) Å
*c* = 16.0377 (4) Å
*V* = 1757.25 (9) Å^3^
*Z* = 4
Mo *K*α radiation
μ = 0.07 mm^−1^
*T* = 293 (2) K
0.30 × 0.24 × 0.20 mm

#### Data collection

Bruker Kappa APEX2 diffractometer
Absorption correction: multi-scan (Blessing, 1995 ▶) *T*_min_ = 0.978, *T*_max_ = 0.985
14793 measured reflections
3559 independent reflections
2611 reflections with *I* > 2σ(*I*)
*R*_int_ = 0.026

#### Refinement

*R*[*F* ^2^ > 2σ(*F* ^2^)] = 0.050
*wR*(*F* ^2^) = 0.141
*S* = 1.09
3559 reflections
226 parameters
H-atom parameters constrained
Δρ_max_ = 0.31 e Å^−3^
Δρ_min_ = −0.21 e Å^−3^

### 

Data collection: *APEX2* (Bruker, 2004 ▶); cell refinement: *APEX2* and *SAINT* (Bruker, 2004 ▶); data reduction: *SAINT* and *XPREP* (Bruker, 2004 ▶); program(s) used to solve structure: *SHELXS97* (Sheldrick, 2008 ▶); program(s) used to refine structure: *SHELXL97* (Sheldrick, 2008 ▶); molecular graphics: *ORTEP-3* (Farrugia, 1997 ▶); software used to prepare material for publication: *PLATON* (Spek, 2003 ▶).

## Supplementary Material

Crystal structure: contains datablocks I, global. DOI: 10.1107/S1600536808009446/gw2038sup1.cif

Structure factors: contains datablocks I. DOI: 10.1107/S1600536808009446/gw2038Isup2.hkl

Additional supplementary materials:  crystallographic information; 3D view; checkCIF report

## Figures and Tables

**Table 1:** Hydrogen-bond geometry (Å, °)

*D*—H⋯*A*	*D*—H	H⋯*A*	*D*⋯*A*	*D*—H⋯*A*
C12—H12*A*⋯N1	0.93	2.59	2.905 (4)	100
C10—H10*A*⋯O1^i^	0.97	2.49	3.446 (3)	170
C23—H23⋯O1^ii^	0.93	2.55	3.356 (3)	146

Symmetry codes: (i) 
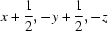
; (ii) 
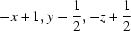
.

**Fig. 1.**
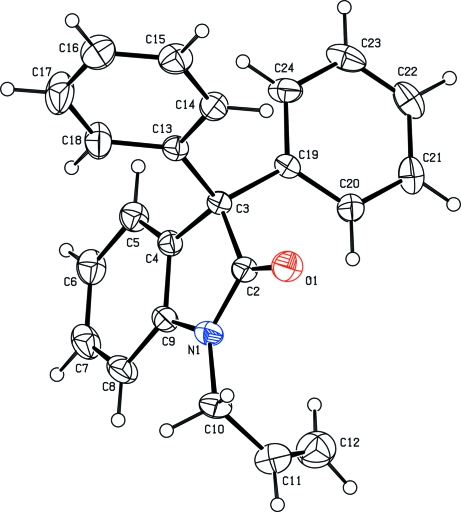
The molecular structure of (I) with 30% probability displacement ellipsoids

**Fig. 2.**
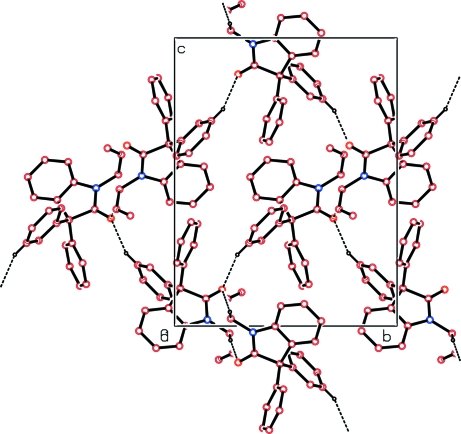
The packing of the molecules viewed down a axis.

## supplementary crystallographic information

### Comment

Indole and its derivatives form a class of toxic recalcitrant N – heterocyclic compounds that are considered as pollutants (Florin *et al.*, 1980). Derivatives of indole have received much attention because of their widespread applications in materials science, agrichemicals, and pharmaceuticals (Ramirez & Garcia-Rubio, 2003).Their preparation and functionalization continues to be a fascinating subject in organic synthesis due to the frequent appearance of indoles in biologically interesting compounds (Bandini *et al.*, 2005). Compounds containing the indole moiety have been proven to display high activity of aldose reductase inhibition (Rajeswaran *et al.*, 1999). In view of this importance, an X-ray study of the title compound, (I), was carried out.

An *ORTEP *(Farrugia,1997) plot of the molecule is shown in Fig.1. The indole moiety (N1/C2 – C9) is planar with the maximum deviation of 0.040 (8) for C2 atom. The oxindole ring is nearly orthogonal to phenyl rings A (C13 – C18) and B (C19 – C24), and makes a dihedral angle of 72.1 (6)° with the ring A, 77.6 (6)° with the ring B. Both the phenyl rings A and B are oriented at an angle of 70.4 (7)° with respect to each other. The sum of angles at N1 [360.0]° indicate *sp*^2 ^hybridization. In the oxindole ring systems, the variation in endocyclic angles are due to fusion of five and six membered rings (Govind *et al.*, 2003). The N1— C2 and C2— O1 bond lengths indicate electron delocalization over atoms N1, C2 and O1. The torsion angle C12—C11—C10—N1 = – 0.3 (4)° indicate that the allyl group deviates significantly from the plane of the attached indole moiety.

Weak intramolecular C—H···N and C—H··· π interactions stabilize the molecule. The crystal packing also involves inter molecular C—H···O interactions and van der Waals forces.

### Experimental

To a solution of phenyl magnesium bromide in dry THF, at 0°C under N_2_ atm.,1-*N*-allyl isatin (0.005 mol, 0.935 g), in dry THF, was added dropwise. After the complete addition, the mixture was stirred at 0°C for 1 hr and then it was stirred at room temperature for 5 hrs. The progress of the reaction was followed by TLC. On completion of the reaction, a saturated solution of NH_4_Cl was added slowly at 0°C. The aqueous layer was washed with brine (50 ml) and dried. On removal of the solvent under reduced pressure, a crude mass was obtained., which was purified over a column of silica gel (100–200 mesh) using hexane/ethyl acetate as eluent. Compound was recrystallized from methanol.

### Refinement

H atoms were placed in idealized positions and allowed to ride on their parent atoms with aromatic C—H distances of 0.93 Å, methylene C—H distances of 0.97 Å and with *U*_iso_(H) = 1.2 *U*_eq_(C).In the absence of significant anomalous scattering effects, Friedel pairs have been merged. The absolute structure parameter is removed from the CIF since the Flack [10.0 (10)] is meaningless.

### Crystal data

**Table:**

C_23_H_19_NO	*F*_000_ = 688
*M**_r_* = 325.39	*D*_x_ = 1.230 Mg m^−^^3^
Orthorhombic, *P*2_1_2_1_2_1_	Mo *K*α radiation λ = 0.71073 Å
Hall symbol: p 2ac 2ab	Cell parameters from 4984 reflections
*a* = 8.8449 (3) Å	θ = 2.5–30.3º
*b* = 12.3879 (4) Å	µ = 0.08 mm^−^^1^
*c* = 16.0377 (4) Å	*T* = 293 (2) K
*V* = 1757.25 (9) Å^3^	Prism, yellow
*Z* = 4	0.30 × 0.24 × 0.20 mm

### Data collection

**Table:**

Bruker Kappa APEX2 diffractometer	3559 independent reflections
Radiation source: fine-focus sealed tube	2611 reflections with *I* > 2σ(*I*)
Monochromator: graphite	*R*_int_ = 0.026
*T* = 293(2) K	θ_max_ = 32.6º
ω and φ scans	θ_min_ = 2.5º
Absorption correction: multi-scan(Blessing, 1995)	*h* = −13→11
*T*_min_ = 0.978, *T*_max_ = 0.985	*k* = −18→14
14793 measured reflections	*l* = −20→24

### Refinement

**Table:**

Refinement on *F*^2^	Secondary atom site location: difference Fourier map
Least-squares matrix: full	Hydrogen site location: inferred from neighbouring sites
*R*[*F*^2^ > 2σ(*F*^2^)] = 0.050	H-atom parameters constrained
*wR*(*F*^2^) = 0.142	*w* = 1/[σ^2^(*F*_o_^2^) + (0.0653*P*)^2^ + 0.2107*P*] where *P* = (*F*_o_^2^ + 2*F*_c_^2^)/3
*S* = 1.09	(Δ/σ)_max_ < 0.001
3559 reflections	Δρ_max_ = 0.31 e Å^−^^3^
226 parameters	Δρ_min_ = −0.21 e Å^−^^3^
Primary atom site location: structure-invariant direct methods	Extinction correction: none

### Special details

**Table:**

Geometry. All e.s.d.'s (except the e.s.d. in the dihedral angle between two l.s. planes) are estimated using the full covariance matrix. The cell e.s.d.'s are taken into account individually in the estimation of e.s.d.'s in distances, angles and torsion angles; correlations between e.s.d.'s in cell parameters are only used when they are defined by crystal symmetry. An approximate (isotropic) treatment of cell e.s.d.'s is used for estimating e.s.d.'s involving l.s. planes.
Refinement. Refinement of *F*^2^ against ALL reflections. The weighted *R*-factor *wR* and goodness of fit *S* are based on *F*^2^, conventional *R*-factors *R* are based on *F*, with *F* set to zero for negative *F*^2^. The threshold expression of *F*^2^ > σ(*F*^2^) is used only for calculating *R*-factors(gt) *etc*. and is not relevant to the choice of reflections for refinement. *R*-factors based on *F*^2^ are statistically about twice as large as those based on *F*, and *R*- factors based on ALL data will be even larger.

### Fractional atomic coordinates and isotropic or equivalent isotropic displacement parameters (Å^2^)

**Table:**

	*x*	*y*	*z*	*U*_iso_*/*U*_eq_	
C2	0.7623 (2)	0.14099 (15)	0.09508 (12)	0.0367 (4)	
C3	0.7846 (2)	0.02597 (14)	0.13122 (10)	0.0331 (3)	
C4	0.8751 (2)	−0.02725 (15)	0.06249 (11)	0.0349 (4)	
C5	0.9240 (3)	−0.13195 (17)	0.05340 (13)	0.0454 (5)	
H5	0.9017	−0.1836	0.0937	0.055*	
C6	1.0075 (3)	−0.1595 (2)	−0.01711 (14)	0.0523 (5)	
H6	1.0420	−0.2299	−0.0239	0.063*	
C7	1.0390 (3)	−0.0829 (2)	−0.07656 (14)	0.0570 (6)	
H7	1.0952	−0.1025	−0.1231	0.068*	
C8	0.9897 (3)	0.0220 (2)	−0.06920 (13)	0.0517 (5)	
H8	1.0119	0.0733	−0.1097	0.062*	
C9	0.9059 (2)	0.04807 (16)	0.00085 (11)	0.0387 (4)	
C10	0.8516 (3)	0.24727 (19)	−0.02570 (15)	0.0543 (6)	
H10A	0.9527	0.2513	−0.0490	0.065*	
H10B	0.8404	0.3073	0.0126	0.065*	
C11	0.7411 (3)	0.2612 (2)	−0.09456 (17)	0.0675 (7)	
H11	0.7486	0.3252	−0.1246	0.081*	
C12	0.6406 (4)	0.1988 (3)	−0.1170 (2)	0.0792 (9)	
H12A	0.6272	0.1334	−0.0894	0.095*	
H12B	0.5781	0.2170	−0.1614	0.095*	
C13	0.8731 (2)	0.03991 (15)	0.21273 (11)	0.0363 (4)	
C14	0.8029 (3)	0.08647 (16)	0.28170 (13)	0.0439 (4)	
H14	0.7018	0.1067	0.2784	0.053*	
C15	0.8814 (3)	0.10282 (19)	0.35467 (14)	0.0534 (6)	
H15	0.8327	0.1333	0.4004	0.064*	
C16	1.0312 (3)	0.0745 (2)	0.36051 (15)	0.0643 (7)	
H16	1.0845	0.0863	0.4097	0.077*	
C17	1.1015 (3)	0.0285 (3)	0.29290 (17)	0.0782 (9)	
H17	1.2028	0.0090	0.2964	0.094*	
C18	1.0225 (3)	0.0110 (3)	0.21964 (15)	0.0597 (6)	
H18	1.0713	−0.0208	0.1745	0.072*	
C19	0.6328 (2)	−0.03049 (15)	0.14321 (11)	0.0347 (4)	
C20	0.5121 (2)	−0.00927 (18)	0.09117 (14)	0.0446 (5)	
H20	0.5221	0.0426	0.0496	0.054*	
C21	0.3761 (3)	−0.0640 (2)	0.09980 (17)	0.0548 (6)	
H21	0.2957	−0.0484	0.0645	0.066*	
C22	0.3604 (3)	−0.1413 (2)	0.16061 (17)	0.0572 (6)	
H22	0.2690	−0.1775	0.1669	0.069*	
C23	0.4792 (3)	−0.1650 (2)	0.21185 (15)	0.0596 (6)	
H23	0.4688	−0.2178	0.2526	0.072*	
C24	0.6155 (3)	−0.11038 (18)	0.20325 (13)	0.0500 (5)	
H24	0.6961	−0.1275	0.2381	0.060*	
N1	0.8393 (2)	0.14731 (13)	0.02162 (10)	0.0414 (4)	
O1	0.69142 (19)	0.21379 (12)	0.12636 (10)	0.0505 (4)	

### Atomic displacement parameters (Å^2^)

**Table:**

	*U*^11^	*U*^22^	*U*^33^	*U*^12^	*U*^13^	*U*^23^
C2	0.0361 (9)	0.0371 (9)	0.0367 (8)	−0.0015 (7)	0.0006 (7)	0.0024 (7)
C3	0.0331 (8)	0.0371 (8)	0.0290 (7)	0.0005 (7)	0.0027 (6)	0.0012 (7)
C4	0.0326 (8)	0.0408 (9)	0.0313 (8)	−0.0012 (7)	0.0004 (7)	−0.0026 (7)
C5	0.0469 (11)	0.0442 (10)	0.0452 (10)	0.0025 (9)	0.0027 (9)	−0.0034 (9)
C6	0.0480 (12)	0.0570 (12)	0.0518 (12)	0.0076 (10)	0.0008 (10)	−0.0170 (10)
C7	0.0455 (12)	0.0809 (17)	0.0446 (11)	−0.0002 (11)	0.0090 (10)	−0.0169 (11)
C8	0.0486 (12)	0.0700 (14)	0.0363 (10)	−0.0056 (11)	0.0102 (9)	0.0001 (10)
C9	0.0349 (9)	0.0463 (9)	0.0351 (8)	−0.0051 (8)	0.0027 (7)	0.0006 (8)
C10	0.0559 (13)	0.0514 (11)	0.0557 (13)	−0.0076 (10)	0.0055 (11)	0.0204 (11)
C11	0.0746 (17)	0.0706 (17)	0.0574 (15)	0.0029 (15)	−0.0022 (14)	0.0130 (13)
C12	0.088 (2)	0.084 (2)	0.0650 (17)	0.0092 (19)	0.0007 (16)	−0.0026 (15)
C13	0.0369 (9)	0.0391 (9)	0.0329 (8)	−0.0001 (7)	0.0011 (7)	0.0000 (7)
C14	0.0458 (11)	0.0473 (10)	0.0386 (10)	0.0052 (9)	0.0013 (8)	−0.0040 (8)
C15	0.0651 (15)	0.0582 (13)	0.0369 (10)	0.0023 (12)	0.0004 (10)	−0.0089 (9)
C16	0.0631 (16)	0.0861 (18)	0.0437 (12)	−0.0075 (14)	−0.0126 (11)	−0.0102 (12)
C17	0.0463 (13)	0.126 (3)	0.0618 (15)	0.0108 (17)	−0.0163 (12)	−0.0218 (18)
C18	0.0395 (11)	0.0917 (18)	0.0479 (11)	0.0075 (12)	−0.0028 (9)	−0.0165 (12)
C19	0.0354 (9)	0.0368 (8)	0.0319 (8)	−0.0017 (7)	0.0050 (6)	0.0008 (7)
C20	0.0394 (10)	0.0457 (10)	0.0489 (11)	0.0001 (8)	0.0001 (8)	0.0063 (9)
C21	0.0343 (10)	0.0569 (13)	0.0730 (15)	−0.0010 (9)	−0.0001 (10)	−0.0082 (12)
C22	0.0471 (12)	0.0530 (12)	0.0713 (15)	−0.0151 (10)	0.0185 (11)	−0.0132 (12)
C23	0.0773 (17)	0.0548 (13)	0.0468 (11)	−0.0253 (12)	0.0135 (12)	0.0028 (10)
C24	0.0602 (14)	0.0513 (12)	0.0387 (10)	−0.0141 (11)	−0.0024 (9)	0.0105 (9)
N1	0.0439 (9)	0.0401 (8)	0.0401 (8)	−0.0042 (7)	0.0052 (7)	0.0081 (7)
O1	0.0565 (9)	0.0411 (7)	0.0539 (9)	0.0091 (7)	0.0066 (7)	0.0007 (6)

### Geometric parameters (Å, °)

**Table:**

C2—O1	1.207 (2)	C12—H12B	0.9300
C2—N1	1.363 (2)	C13—C18	1.374 (3)
C2—C3	1.551 (3)	C13—C14	1.393 (3)
C3—C4	1.513 (2)	C14—C15	1.376 (3)
C3—C19	1.526 (3)	C14—H14	0.9300
C3—C13	1.533 (2)	C15—C16	1.374 (4)
C4—C5	1.375 (3)	C15—H15	0.9300
C4—C9	1.387 (3)	C16—C17	1.373 (4)
C5—C6	1.393 (3)	C16—H16	0.9300
C5—H5	0.9300	C17—C18	1.384 (3)
C6—C7	1.374 (3)	C17—H17	0.9300
C6—H6	0.9300	C18—H18	0.9300
C7—C8	1.375 (4)	C19—C20	1.380 (3)
C7—H7	0.9300	C19—C24	1.389 (3)
C8—C9	1.384 (3)	C20—C21	1.388 (3)
C8—H8	0.9300	C20—H20	0.9300
C9—N1	1.404 (3)	C21—C22	1.374 (4)
C10—N1	1.456 (3)	C21—H21	0.9300
C10—C11	1.485 (4)	C22—C23	1.366 (4)
C10—H10A	0.9700	C22—H22	0.9300
C10—H10B	0.9700	C23—C24	1.390 (3)
C11—C12	1.232 (4)	C23—H23	0.9300
C11—H11	0.9300	C24—H24	0.9300
C12—H12A	0.9300		
			
O1—C2—N1	125.14 (18)	C18—C13—C14	118.21 (19)
O1—C2—C3	126.65 (17)	C18—C13—C3	122.03 (18)
N1—C2—C3	108.20 (15)	C14—C13—C3	119.73 (17)
C4—C3—C19	110.95 (15)	C15—C14—C13	120.7 (2)
C4—C3—C13	113.58 (15)	C15—C14—H14	119.6
C19—C3—C13	113.16 (14)	C13—C14—H14	119.6
C4—C3—C2	101.25 (14)	C16—C15—C14	120.5 (2)
C19—C3—C2	110.88 (15)	C16—C15—H15	119.8
C13—C3—C2	106.26 (14)	C14—C15—H15	119.8
C5—C4—C9	119.81 (17)	C17—C16—C15	119.2 (2)
C5—C4—C3	130.89 (17)	C17—C16—H16	120.4
C9—C4—C3	109.29 (16)	C15—C16—H16	120.4
C4—C5—C6	118.9 (2)	C16—C17—C18	120.5 (2)
C4—C5—H5	120.5	C16—C17—H17	119.8
C6—C5—H5	120.5	C18—C17—H17	119.8
C7—C6—C5	120.1 (2)	C13—C18—C17	120.8 (2)
C7—C6—H6	119.9	C13—C18—H18	119.6
C5—C6—H6	119.9	C17—C18—H18	119.6
C6—C7—C8	121.9 (2)	C20—C19—C24	118.03 (18)
C6—C7—H7	119.0	C20—C19—C3	121.12 (16)
C8—C7—H7	119.0	C24—C19—C3	120.69 (18)
C7—C8—C9	117.4 (2)	C19—C20—C21	121.1 (2)
C7—C8—H8	121.3	C19—C20—H20	119.4
C9—C8—H8	121.3	C21—C20—H20	119.4
C8—C9—C4	121.79 (19)	C22—C21—C20	119.9 (2)
C8—C9—N1	128.49 (18)	C22—C21—H21	120.0
C4—C9—N1	109.72 (16)	C20—C21—H21	120.0
N1—C10—C11	115.9 (2)	C23—C22—C21	119.9 (2)
N1—C10—H10A	108.3	C23—C22—H22	120.0
C11—C10—H10A	108.3	C21—C22—H22	120.0
N1—C10—H10B	108.3	C22—C23—C24	120.2 (2)
C11—C10—H10B	108.3	C22—C23—H23	119.9
H10A—C10—H10B	107.4	C24—C23—H23	119.9
C12—C11—C10	128.2 (3)	C19—C24—C23	120.7 (2)
C12—C11—H11	115.9	C19—C24—H24	119.6
C10—C11—H11	115.9	C23—C24—H24	119.6
C11—C12—H12A	120.0	C2—N1—C9	111.39 (15)
C11—C12—H12B	120.0	C2—N1—C10	122.46 (18)
H12A—C12—H12B	120.0	C9—N1—C10	126.12 (17)
			
O1—C2—C3—C4	177.4 (2)	C3—C13—C14—C15	−177.99 (18)
N1—C2—C3—C4	−3.70 (18)	C13—C14—C15—C16	0.7 (4)
O1—C2—C3—C19	59.6 (2)	C14—C15—C16—C17	−0.7 (4)
N1—C2—C3—C19	−121.48 (16)	C15—C16—C17—C18	0.1 (5)
O1—C2—C3—C13	−63.8 (2)	C14—C13—C18—C17	−0.6 (4)
N1—C2—C3—C13	115.17 (16)	C3—C13—C18—C17	177.3 (3)
C19—C3—C4—C5	−57.8 (3)	C16—C17—C18—C13	0.6 (5)
C13—C3—C4—C5	71.0 (3)	C4—C3—C19—C20	−80.4 (2)
C2—C3—C4—C5	−175.6 (2)	C13—C3—C19—C20	150.58 (17)
C19—C3—C4—C9	120.96 (17)	C2—C3—C19—C20	31.3 (2)
C13—C3—C4—C9	−110.24 (17)	C4—C3—C19—C24	95.0 (2)
C2—C3—C4—C9	3.23 (18)	C13—C3—C19—C24	−34.0 (2)
C9—C4—C5—C6	1.6 (3)	C2—C3—C19—C24	−153.30 (18)
C3—C4—C5—C6	−179.68 (19)	C24—C19—C20—C21	1.6 (3)
C4—C5—C6—C7	−0.5 (3)	C3—C19—C20—C21	177.17 (19)
C5—C6—C7—C8	−0.3 (4)	C19—C20—C21—C22	−0.5 (4)
C6—C7—C8—C9	−0.2 (4)	C20—C21—C22—C23	−0.7 (4)
C7—C8—C9—C4	1.4 (3)	C21—C22—C23—C24	0.6 (4)
C7—C8—C9—N1	−177.8 (2)	C20—C19—C24—C23	−1.7 (3)
C5—C4—C9—C8	−2.2 (3)	C3—C19—C24—C23	−177.3 (2)
C3—C4—C9—C8	178.88 (19)	C22—C23—C24—C19	0.6 (4)
C5—C4—C9—N1	177.21 (18)	O1—C2—N1—C9	−178.1 (2)
C3—C4—C9—N1	−1.7 (2)	C3—C2—N1—C9	3.0 (2)
N1—C10—C11—C12	−0.3 (4)	O1—C2—N1—C10	3.8 (3)
C4—C3—C13—C18	3.0 (3)	C3—C2—N1—C10	−175.20 (18)
C19—C3—C13—C18	130.7 (2)	C8—C9—N1—C2	178.5 (2)
C2—C3—C13—C18	−107.4 (2)	C4—C9—N1—C2	−0.8 (2)
C4—C3—C13—C14	−179.13 (17)	C8—C9—N1—C10	−3.4 (4)
C19—C3—C13—C14	−51.5 (2)	C4—C9—N1—C10	177.25 (19)
C2—C3—C13—C14	70.4 (2)	C11—C10—N1—C2	−96.5 (3)
C18—C13—C14—C15	−0.1 (3)	C11—C10—N1—C9	85.6 (3)

### Hydrogen-bond geometry (Å, °)

**Table:**

*D*—H···*A*	*D*—H	H···*A*	*D*···*A*	*D*—H···*A*
C12—H12A···N1	0.93	2.59	2.905 (4)	100
C10—H10A···O1^i^	0.97	2.49	3.446 (3)	170
C23—H23···O1^ii^	0.93	2.55	3.356 (3)	146

Symmetry codes: (i) *x*+1/2, −*y*+1/2, −*z*; (ii) −*x*+1, *y*−1/2, −*z*+1/2.
